# Fatigue as hallmark of Fabry disease: role of bioenergetic alterations

**DOI:** 10.3389/fcvm.2024.1341590

**Published:** 2024-01-24

**Authors:** Jessica Gambardella, Eleonora Riccio, Antonio Bianco, Antonella Fiordelisi, Federica Andrea Cerasuolo, Antonietta Buonaiuto, Teodolinda Di Risi, Alessandro Viti, Roberta Avvisato, Antonio Pisani, Daniela Sorriento, Guido Iaccarino

**Affiliations:** ^1^ Centro Interdipartimentale di Ricerca in Ipertensione Arteriosa e Patologie Associate, Federico II University of Naples, Naples, Italy; ^2^ Federico II University Hospital, Naples, Italy; ^3^ CEINGE “Franco Salvatore”, Naples, Italy

**Keywords:** Fabry, metabolism, fatigue, skeletal muscle, exercise intolerance

## Abstract

Fabry disease (FD) is a lysosomal storage disorder due to the impaired activity of the *α*-galactosidase A (GLA) enzyme which induces Gb3 deposition and multiorgan dysfunction. Exercise intolerance and fatigue are frequent and early findings in FD patients, representing a self-standing clinical phenotype with a significant impact on the patient's quality of life. Several determinants can trigger fatigability in Fabry patients, including psychological factors, cardiopulmonary dysfunctions, and primary alterations of skeletal muscle. The “metabolic hypothesis” to explain skeletal muscle symptoms and fatigability in Fabry patients is growing acknowledged. In this report, we will focus on the primary alterations of the motor system emphasizing the role of skeletal muscle metabolic disarrangement in determining the altered exercise tolerance in Fabry patients. We will discuss the most recent findings about the metabolic profile associated with Fabry disease offering new insights for diagnosis, management, and therapy.

## 1 Introduction

Fabry disease (FD) is an inherited X-linked lysosomal storage disorder due to the impaired activity of the *α*-galactosidase A (GLA) enzyme. The partial or total inactivity of this enzyme, essential to metabolize glycosphingolipids (GB3), induces the deposition of GB3 in lysosomes of cells in several organs and tissues evolving progressively toward multiorgan dysfunction (1, 2). The molecular pathogenesis of FD encompasses several pathologic mechanisms involving mitochondrial dysfunction, lysosomal dysfunction, GB3 accumulation, endothelial dysfunction, and autophagy abnormalities (3, 4). Today, more than 1,000 GLA gene variants have been identified in the chromosomal region Xq22.1, including splicing alterations, deletions, translocations, complex gene rearrangement, and point missense variants (5, 6), but an exact genotype-phenotype correlation in FD cannot be established. The signs and symptoms of FD are heterogeneous with high variability among patients (7). Males carrying the defective gene will develop the pathology with often severe clinical manifestations while females, once thought to be just asymptomatic carriers, could develop the disease with mild to severe signs (8). The complete loss of enzyme function is usually associated with the “classic” phenotype with severe symptoms appearing in childhood while the residual enzyme activity may lead to a slow progression of the disease (the “late-onset” phenotype) with milder symptoms occurring in adults. Pain, and gastrointestinal (9) ocular, ear, or skeletal manifestations are the earliest symptoms of Fabry disease, which are not always immediately associated with FD. If left untreated, this multisystemic disease is progressive with cardiac and renal involvements as severe complications (10–12). FD may also manifest with mild nonspecific symptoms frequently affecting the musculoskeletal system (13).

## 2 Therapies and management of FD

As a multiorgan pathology, FD management requires multidisciplinary care involving cardiology, nephrology, gastroenterology, neurology, psychology, and genetics, aiming to treat the specific symptoms by available current therapies, ameliorate the quality of life, manage pain, and give psychosocial support. Guidelines for the diagnosis and treatment of FD are available for both adults (14, 15) and children (16), establishing the inclusion and exclusion criteria for treatments. The disease presents a variable expression of symptoms, which are also gender and age-dependent, therefore, their association with FD is very difficult. To identify Fabry patients and avoid delay or lack of diagnosis, it is essential to consider all potential conditions that could generate “clinical suspicion” and trigger the diagnostic process as soon as possible.

The FD diagnosis is mainly based on the genetic screening of GLA gene mutations and activity, especially for women as enzymatic levels of the female heterozygote could not associate with the pathologic manifestations. Such diagnosis is then confirmed by the analysis of urine Gb3 and plasma lyso-Gb3 levels.

Available treatments are based on the administration of intravenous/oral specific drugs and adjuvant therapies (renal, cardiac, neurological therapies) aiming to treat symptoms and prevent the progression toward organ complications. The enzymatic replacement therapy (ERT) with agalsidase alfa (Replagal) and agalsidase beta (Fabrazyme), was the first to be identified in 2001 and is administered once every 2 weeks by intravenous injections. Clinical data suggest that treatment is improved with early ERT initiation (12, 13). The oral chaperone Migalastat was approved in Europe in 2016 for the treatment of patients carrying amenable mutations. It is a well-tolerated therapy that can be administered orally every other day with a promising impact on both primary and secondary endpoints of FD.

## 3 Exercise intolerance and fatigue in FD: the clinical relevance

Fatigue is the impairment of the ability to carry out the usual daily activities, and is different from muscle weakness, due to myopathy or a neurological disorder. The main symptoms of fatigue are listlessness, lack of energy, exhaustion, tiredness, early fatigability, sleepiness, a tendency to fall asleep during the day, physical weakness, or a feeling of running on empty. Fatigue represents the most bothersome symptom for most pathological conditions, including Fabry disease.

Exercise intolerance and fatigue are frequent and early findings in FD patients and were previously considered a consequence of diastolic heart failure or related to peripheral nerve involvement. Today, several reports suggest that the reduced tolerance to physical activity in patients with FD is not a secondary effect of diastolic heart failure, but a self-standing clinical phenotype that occurs independently from chronic renal or cardiac dysfunction (17–19). The involvement of the skeletal muscle leads to muscular cramps in the early phase and muscular pain, fatigability, and asthenia, in the advanced phases of the disease which involve walking and motor performance (19). The resulting chronic fatigue has a significant impact on quality of life, affecting patients' daily activities. This mainly concerns children's participation in after-school activities, and alterations of concentration and memory at work, with resulting negative impacts also on psychosocial attitudes.

## 4 Fatigue management

Since FD is a multiorgan disease, the treatment is focused not only on reactivating the enzymatic activity of GLA but also on the specific management of symptoms, which vary among patients. Chronic fatigue occurs in over half of FD patients but is also the most difficult FD symptom to manage since current therapies are not effective in reducing fatigue and ameliorating the quality of life (20). Moreover, no alternative treatments are available considering the scarce knowledge of its pathogenetic mechanism. Pain medications and changes in lifestyles, including stress reduction and exercise, could ameliorate energy levels.

A cardiopulmonary exercise test is a good tool to evaluate cardiac function and aerobic fitness and to determine how the heart responds to exercise-induced stress (21) but despite muscular symptoms in FD, just a few studies evaluated cardiopulmonary fitness with exercise testing in Fabry patients (21–24). This test is instead essential to assess functional capacity and aerobic fitness level and could also be useful to perform disease diagnosis, determine symptoms severity, and monitor the effects of treatments, including exercise programs. The few recent findings suggest that Fabry patients have impaired cardiopulmonary exercise capacity and failed to reach maximum heart rate both before and after ERT treatment (21). This suggests that alterations in the exercise performance of Fabry patients cannot be improved by ERT. Similarly, Migalastat therapy cannot revert the muscular phenotype in FD even if an 18-month treatment was associated with a “trend” towards an improvement in exercise tolerance (25). In our cohort of Fabry patients, we observed a significant reduction of aerobic exercise tolerance alongside an accumulation of stress-induced lactate production (26). Interestingly, this phenomenon occurs also in young and female patients, in which the classical Fabry phenotypes were not still obvious (kidney and cardiac symptoms). Moreover, in a small pool of our FD population, we retrospectively tested the effects on exercise tolerance of available FD therapies. After one year, in non-treated patients, exercise duration was comparable to the baseline, whereas in the treated subgroup we observed a significant improvement in exercise endurance (26). This finding suggests that the reduced tolerance to exercise is a specific and sensitive hallmark of FD which might be included in the panel of clinical parameters useful to establish the starting point of the therapy and to monitor its efficacy. In this perspective, exercise-induced blood lactate accumulation could also be considered a potentially useful biomarker for monitoring muscular phenotype in Fabry patients. Nevertheless, further dedicated studies are needed to definitely establish the benefits of available FD therapies on fatigue and exercise intolerance in FD patients.

In some cases, exercise intolerance in FD patients could result from physical inactivity suggesting the clinical benefits of an exercise program (27), and the possibility to include FD in the plethora of chronic pathologies within the Adapted Physical Activity (AFA) programs. However, there are no specific guidelines for exercise prescription in the FD population. Certainly, the complexity of the phenotype requires a thorough assessment of the clinical phenotype before prescription. The general guidelines for exercise and physical activity by the American College of Sports Medicine (ACSM) might be adapted to the FD population as previously tested by Schmitz and colleagues (27). Specifically, they evaluated the effects of an adapted strength/circuit exercise program on a small population of FD patients. The results indicated that regular training for 12 months was able to improve exercise capacity, muscle strength, and the general well-being of FD participants. The study by Schmitz et al. is the only one to present data on a prospective training intervention in patients with FD. Hence, there is an urgent need for further studies exploring the effects of exercise programs on a larger FD population. With more data, it will be possible to draft a specific FD-AFA program and also to establish the impact of disease severity in order to determine which FD patients will benefit from certain training interventions.

## 5 Pathogenetic mechanisms of fatigue in FD patients

Fatigue is a very complex symptom and, as such, it is highly subjective and difficult to define, evaluate, and quantify. Broadly, fatigue can be described as an overwhelming sense of tiredness, lack of energy, and feeling of exhaustion (28, 29). Despite the high impact on patient's quality of life, fatigue is an under-recognized and undertreated condition (30). It is a common feature of a wide variety of disorders including infective, neurological, psychiatric, neoplastic, metabolic diseases, and myopathies (31–33). Fatigue can be classified as “mental fatigue”, which refers to the cognitive or perceptual aspects of fatigue, and “physical” or muscular fatigue, which refers to the performance of the motor system (34). Specifically, muscle fatigue is defined as the inability of muscles to generate an expected power or to maintain the required force for a given task (35). Muscle fatigue, in turn, can be distinguished in central or peripheral according to which level of the motor system is affected. Central fatigue originates in the central nervous system (CNS), with a decreased neural drive to the muscle. Peripheral fatigue arises from the muscle and predominately involves muscle bioenergetics or excitation-contraction mechanism alterations.

Fatigue is commonly experienced by Fabry patients and despite the exact pathogenetic mechanisms remain unknown, different components are suggested to contribute to its generation. Cognitive impairment and psychological components like depressive symptoms have been associated with pain, negative health perception, and “mental fatigue” in Fabry patients (36). However, “muscle fatigue” in Fabry disease seems to be more relevant and it is attracting a growing interest. Organ damage could surely contribute to chronic muscle fatigue, considering that kidney and heart damage can lead to anemia, and lung alterations can affect breathing (37). However, several Fabry patients without organ complications still suffer from muscle fatigue (17) suggesting that the motor system is primarily affected and muscle fatigue is an independent symptom in FD.

**Figure 1:**
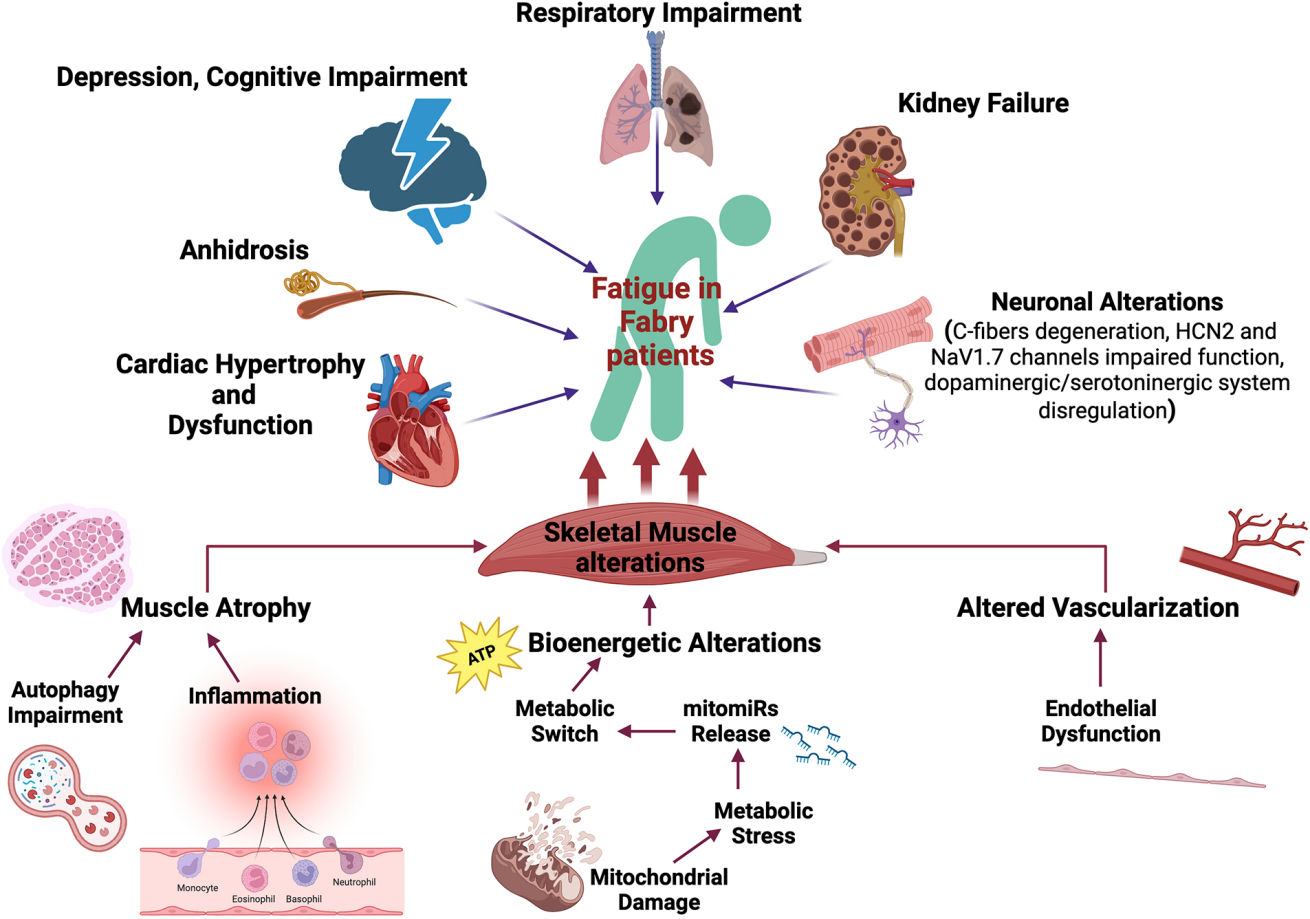
Fatigue determinants in Fabry disease. Several mechanisms are potentially involved in the fatigability experienced by Fabry patients including cardiopulmonary alterations, kidney failure, anhidrosis, cognitive impairment, and skeletal muscle disarrangement. Specifically, primarily skeletal muscle alterations that can strongly impact physical performance have been described: (i) inflammation can potentially drive muscle atrophy; (ii) endothelial dysfunction can induce altered muscle vascularization with impaired oxygen and nutrient supply; (iii) putative mitochondrial dysfunction can induce bioenergetic alterations. The metabolic stress triggers the release of specific mitomiRs which can drive a maladaptive metabolic reprogramming of skeletal muscle. The illustration has been created by Biorender (License number EW26AUO200).

Very little data has been published regarding the mechanisms altered in Fabry disease and their specific impact on chronic fatigue. In the next sections, we provide a summary of the evidence available in the literature, and of their hypothesized clinical implications. Specifically, we will focus on the primary alterations of the motor system, without excluding that other mechanisms could indirectly trigger fatigability in Fabry patients like pulmonary, and cardiac alterations as well as anhidrosis, frequently described in Fabry patients (37–40). Figure 1 summarizes the main contributors to Fatigue in Fabry patients, emphasizing the role of skeletal muscle alterations.

## 6 The “metabolic hypothesis”

The “metabolic hypothesis” to explain skeletal muscle symptoms and fatigability in Fabry patients is growing acknowledged. It takes ground from the biochemistry of the Fabry Muscle which appears to be very different from that of non-Fabry patients.

### 6.1 Relevance of skeletal muscle bioenergetics

Muscular work must be supported by a high supply of ATP energy. Indeed, skeletal muscle is the district with the highest energetic demand, and as such, it is the main determinant of the whole body's metabolic rate. In particular, skeletal muscle can vary its metabolism to a greater extent than any other tissue through high metabolic flexibility, shifting its reliance between anaerobic glycolysis with lactate production, and aerobic oxidation of pyruvate or lipid (41, 42). Disruptions of metabolic flexibility of skeletal muscle are associated with several pathological conditions characterized by exercise intolerance and fatigue (42, 43). Growing evidence supports significant metabolic alterations of skeletal muscle in Fabry disease. We have recently shown a muscle fibers switch in a mouse model of Fabry Disease resulting in a higher abundance of Type II/glycolytic fibers. Accordingly, we recorded high glycolytic rate and lactate overproduction, in line with reduced exercise tolerance and fatigability in Fabry mice and patients (26). The mechanism of this metabolic remodeling involves miR-17-mediated HIF-1 upregulation, which in turn induces the high expression of the key enzymes of lactacid metabolism. As we have shown in another recent report, the recruitment of miR17 alongside other metabolism/mitochondria-related miRNAs (mitomiRs) is likely induced by the intrinsic metabolic stress of Fabry cells (44). Specifically, mitomiRs regulating fundamental aspects of mitochondrial homeostasis and fitness, including expression and assembly of the respiratory chain, mitogenesis, antioxidant capacity, and apoptosis are significantly dysregulated in FD patients. Accordingly, oxidative stress biomarkers including [Advanced Oxidation Protein Products (AOPP), Ferric Reducing Antioxidant Power (FRAP), and thiolic groups] are altered in FD patients already at an early stage of the disease (45). This evidence suggests the early involvement of mitochondrial dysfunction as a mechanism of metabolic and bioenergetic stress in Fabry disease, according to the reduced expression of respiratory chain complexes and the decline of ATP production in Fabry patients (44, 46). In this view, the lactacid metabolic switch of Fabry skeletal muscle could represent an adaptative mechanism to the failure of mitochondrial energetic production, a stress response driven by miR17. This phenomenon occurs at the expense of physical performance and fatigability of Fabry patients, thus representing a maladaptive response that requires monitoring and specific treatments.

In chronic fatigue syndrome (CFS), a targeted, broad-spectrum metabolomics performed on plasma from patients has indicated significant abnormalities in 20 metabolic pathways, including sphingolipid, phospholipid, and mitochondrial metabolic pathways (47). This evidence further supports the “metabolic hypothesis” in the explanation of muscle fatigue in Fabry disease. Supporting data was also derived from the metabolomic and lipidomic analysis that we conducted in a Fabry population (26). Specifically, we have shown a metabolic remodeling in Fabry patients characterized by reduced levels of acetyl-carnitine, fatty acids, and diacyl glycerol, alongside triglycerides accumulation, confirming the altered mitochondrial oxidation of lipids. A similar metabolic profile has been described for *metabolic myopathies*, a group of genetic myopathies characterized by an increased rate of anaerobic glycolysis, exercise intolerance, blood lactate accumulation, and reduction of circulating Acylcarnitine (48, 49). Taken together, these considerations request a redefinition of Fabry disease as a metabolic disorder where the skeletal muscle is a key involved district, and exercise intolerance and fatigue are specific and primary symptoms to be managed.

### 6.2 Muscle atrophy

The atrophy of muscle mass could be another reason for compromised physical performance and fatigability in Fabry patients. Indeed, among the primary myopathic changes in FD, areas of muscle atrophy have been described as granules (19, 50, 51). The immobilization, and sedentary lifestyle are important factors inducing muscle atrophy, and loss of strength (52). However, in Fabry disease, more specific mechanisms able to actively trigger myosin loss could be involved. Among them, pro-inflammatory signaling activation with TNF-α and IL-6 production has been indicated as a potent trigger of ubiquitin ATP-dependent proteasome (UPP response) in the skeletal muscle causing muscular loss (53). Fabry disease was associated with elevated serum levels of IL-6 and TNF-α, and their levels strongly correlated with MSSI scores reflecting a greater disease burden (54).

Lysosomal dysfunction and autophagy dysregulation in Fabry disease could be another mechanism inducing muscle atrophy in Fabry patients. Indeed, autophagy in skeletal muscles is finely tuned under both, physiological conditions and metabolic stress. Altered autophagy activity characterized by either increased formation of autophagosomes or inhibition of lysosome-autophagosome fusion has been identified as one of the major causes of muscle loss in several skeleton muscle disorders (55).

### 6.3 Neuronal contribution

Degeneration of small fibers (C-fibers) and impaired function of ion channels like HCN2 and NaV1.7 sodium channel has been associated with widespread pain and chronic muscle fatigue in Fabry patients, alongside nausea, constipation or diarrhea, and itching (56, 57). Specifically, the pattern of small fiber dysfunction in Fabry disease is similar to other diseases characterized by peripheral neuropathy, including diabetes mellitus (58).

Central neurotransmitters, including serotonin and dopamine, play an important role during whole-body exercise and fatigue. The so-called central fatigue hypothesis states that exercise affects the concentration of these neurotransmitters (within the CNS, or proximal to the neuromuscular junction) thus generating fatigue (59). From a neuroimaging study with 18F-DOPA PET scans, a presynaptic dopaminergic disruption emerges in Fabry patients (60). Moreover, an imbalance between cholinergic and dopaminergic activity in the basal ganglia, which has a central role in movement disorders, has been suggested also for Fabry disease. Alterations of the serotonergic system have been hypothesized as well since a large Gb3 accumulation in serotonergic nuclei of Fabry patients was described (61).

### 6.4 Vasculature and blood flow alterations: reduced O_2_ muscular delivery

An adequate blood flow ensures an adequate oxygen delivery to the working muscle necessary for aerobic ATP production and for removing waste products of metabolic processes, thus playing an important role in the maintenance of force output. Indeed, the occlusion of blood flow to a working muscle induces a reduction of the time to exhaustion and a decline in generated force indicating the importance of blood flow in fatigue prevention (62, 63). Vascular remodeling has been described in Fabry patients. Specifically, a considerable accumulation of glycosphingolipid occurs in vascular smooth muscle and endothelial cells disturbing peripheral endothelial function and promoting intima-media thickening (64). Indeed, high levels of plasmatic endothelial biomarkers have been detected in Fabry patients, like VCAM-1, indicative of considerable vascular dysfunction (65). Inflammation-mediated endothelial dysfunction seems to be also involved; the release of heparanases, a marker of chronic inflammation, appears to be responsible for the degradation of the endothelial glycocalyx, contributing to endothelial dysfunction in FD (66).

Some data suggest a contribution of vasculature alterations in the reduced physical performance and muscle fatigue in Fabry patients. Indeed, a study of histologic examination of Fabry skeletal muscle indicated that muscle myocytes were unaffected, whereas muscle vessels showed the presence of mild glycosphingolipid accumulation in endothelial and smooth muscle cells (19). Moreover, evidence of a perfusion change in the vasa nervorum could also contribute to the dysfunctional processing of sensory information, which likely occurs under physical stress and generates fatigue sensation (67).

## 7 Future directions

FD has a serious impact on the quality of life, morbidity, and mortality of affected people. Great advances have been made in the last decade to identify both early markers of pathology and more effective treatments but several issues in the management of this pathology remain to be solved. First of all, genetic testing for FD should be improved to identify more affected patients and properly start the treatment. The genetic identification of the pathology is just done when the worse complications are manifested and the therapy could be less effective at this stage. Only family members of identified patients are recommended for genetic counseling and benefit from an early diagnosis and treatment. Also, an optimal time to start therapy should be evaluated as well as the possibility of combined therapies to ameliorate patients' quality of life. For chronic fatigue, in particular, specific research is essential to establish the triggering mechanisms and potential specific treatments that could impact both body and mind of Fabry patients. In this view, the cardiopulmonary exercise test and exercise tolerance test should be used as diagnostic and follow-up tools in the clinic management of Fabry patients, to identify exercise intolerance and to monitor the effectiveness of treatments. Also, establishing precise and individual exercise programs for Fabry patients based on their clinical features and exercise tolerance could not only ameliorate physical conditions but also have a psychosocial effect with a great impact on the quality of life. Exercise intolerance is a sensitive and early disease manifestation that could be used also to monitor FD females, which are often undertreated basing the decision on classic phenotypes (cardiac and renal dysfunction). Future studies will be specifically dedicated to FD females evaluating the potential use of exercise testing for monitoring disease stage and for deciding the starting point of the treatment. The assessment of metabolic alterations should also be considered as part of the diagnostic and monitoring iter of Fabry patients. In particular, a panel including lactate, mitomiRs, TG, and Acylcarnitine could be employed to evaluate the entity of metabolic dysregulation, potentially useful to risk stratify Fabry patients and to design a specific therapeutic plan for early intervention. Especially, stress-induced lactate production represents an easy-to-detect, low-cost biomarker of skeletal muscle involvement and muscular metabolic dysregulation, which assessment should attend the exercise tolerance test in Fabry patients routinely.
